# A Randomized Controlled Trial Investigating the Feasibility of a Low-Intensity Psychological Intervention for Fear of Memory Loss and Quality of Life in Older Adults: Protocol for the Reducing Fear and Avoidance of Memory Loss (REFRAME) Study

**DOI:** 10.2196/30514

**Published:** 2021-07-30

**Authors:** Patricia O'Loughlin, Pavithra Pavithra, John Regan, Marc Bennett, Rachel Knight, Bert Lenaert, Melissa Marquez, Michelle Taddeo, James Griffith, Rita Shapiro, Francesca Farina

**Affiliations:** 1 School of Psychology Trinity College Dublin Dublin Ireland; 2 Medical Research Council Cognition and Brain Sciences Unit University of Cambridge United Kingdom; 3 Faculty of Health, Medicine and Life Sciences Limburg Brain Injury Centre Maastricht University Netherlands; 4 Feinberg School of Medicine Northwestern University Chicago, IL United States; 5 VA Chicago Health Care System Chicago, IL United States; 6 Global Brain Health Institute, Trinity College Institute of Neuroscience Trinity College Dublin Dublin 2 Ireland

**Keywords:** fear, memory loss, dementia, older adults, mindfulness, behavioral activation

## Abstract

**Background:**

Dementia is the most feared disease associated with aging. Prolonged fears about memory loss and dementia can have harmful consequences even in the absence of cognitive decline. Fear of dementia is associated with poorer health outcomes and psychological well-being and increased memory failures in older adults.

**Objective:**

We will conduct a randomized controlled trial to determine the feasibility of a tailored, web-based mindfulness program to reduce fear of memory loss and increase quality of life in older adults experiencing heightened fear.

**Methods:**

Eighty participants will be recruited and divided into 2 groups (40 in each group). One group will receive psychoeducation plus mindfulness training. A second group will receive psychoeducation, mindfulness training, and additional modules targeting maladaptive behavioral avoidance (ie, social and cognitive withdrawal).

**Results:**

Our recent etiological model posits that maladaptive behavioral avoidance strategies critically underlie psychosocial dysfunction associated with fear of memory loss. Thus, we predict better outcomes in the second group, including reduced fear of memory loss (primary outcome), Alzheimer disease, anxiety, and subjective memory failures, and increased quality of life (secondary outcomes). Outcome measures will be applied at 5 time points (before, baseline, interim, and after the intervention, and at 3-month follow-up). Data will be analyzed using mixed models and correlations.

**Conclusions:**

Results from this study will contribute to the current literature on dementia-related fear and improve our understanding of how to effectively address and reduce these fears.

**Trial Registration:**

ClinicalTrials.gov NCT04821960; https://clinicaltrials.gov/ct2/show/NCT04821960.

**International Registered Report Identifier (IRRID):**

PRR1-10.2196/30514

## Introduction

### Background

Fear of dementia, also referred to as “dementia worry” [1] and “anticipatory dementia” [2], describes the fear that perceived changes in one’s memory are indicative of dementia [3]. Many older adults worry about developing dementia, with evidence suggesting that dementia has become the most feared health condition among people over the age of 50, ranking higher than cancer and heart disease [4]. The number of people living with dementia worldwide is expected to triple from approximately 50 million to 152 million by 2050. Given this projected increase, the incidence of fear of dementia is also certain to increase [5]. The anticipated growth in dementia cases, coupled with improved societal awareness of the disease and the knowledge that as of yet there is no cure for dementia, has contributed to increased dementia-related fear in older adults [1,6].

Fear of dementia has been associated with poor health outcomes, lower levels of psychological well-being, memory failures, and lower quality of life in older adults [3,7]. Emerging evidence suggests that dementia-related fears perpetuate harmful cognitive–behavioral cycles [3,8,9]; for example, individuals who worry about developing dementia might fixate on what they perceive to be symptoms of neurodegeneration, such as when they forget someone’s name. This excessive self-monitoring is fatiguing and can increase the frequency of cognitive failures, compounding the initial fear. In addition to self-monitoring, individuals can develop unnecessary and unproductive behavioral strategies to mitigate psychological distress and cognitive failures. These maladaptive strategies could include social withdrawal and avoidance of cognitive effort. In a recent study, Farina et al [3] showed that fear and avoidance of memory loss symptoms were associated with lower quality of life and greater self-reported memory failures in a community sample of older adults. Building on this, we posit that psychological distress and everyday cognitive failures may trigger maladaptive behavioral responses that undermine motivation and engagement in healthy activities, which could, in turn, accelerate cognitive decline.

Although many studies have investigated factors associated with dementia-related fear, only few have investigated ways to mitigate this fear. Interventions that disrupt the fear cycle have potential to reduce distress, while also accruing long-term benefits such as preventing the onset of cognitive decline [10]. One promising avenue is mindfulness-based training, which has been shown to improve health and well-being outcomes in other health-related difficulties, such as chronic pain and fatigue [11,12]. These outcomes may be driven by improvements in individuals’ ability to acknowledge negative thoughts and feelings without fixating on them [13-15]. To our knowledge, no studies have investigated the efficacy of mindfulness-based interventions for dementia-related fear. Another promising avenue for tackling dementia-related fear is approach-oriented exercises, in which people practice behaviors that allow them to exercise their cognitive abilities even in the face of emotional distress. Although approach-oriented activities (eg, behavioral activation, exposure therapy) are widespread in behavior therapy, specific techniques for dementia-related fear warrant further exploration [16,17]. Further, a person with a clinically elevated level of dementia-related fear might be referred to clinical care (eg, working with a psychologist or social worker), but scalable interventions for subclinical fears are also needed.

This study will leverage web-based technology to deliver and compare 2 low-intensity, self-guided approaches to help older people manage dementia-related fears in daily life: (1) mindfulness and meditation exercises, and (2) mindfulness and meditation exercises combined with behavioral therapy exercises to facilitate approach, rather than avoidance. This research will contribute to existing knowledge of dementia-related fear and improve our understanding of how to effectively address and reduce these fears.

### Purpose

The purpose of this study is to compare web-based programs to reduce fear of memory loss and increase quality of life in older adults experiencing dementia-related fear. This program (REFRAME) will focus on 3 components across 3 weeks. These include psychoeducation (Week 1), mindfulness-based exercises to identify and monitor dementia-related fears (Week 2), and behavioral activation to overcome unnecessary and maladaptive avoidance behaviors (Week 3). Further, we propose that maladaptive avoidance behaviors are a critical factor in maintaining dementia-related anxiety. Therefore, REFRAME will be tested against an active comparison group receiving psychoeducation and mindfulness only. The overarching goals of the project are to (1) determine the impact of REFRAME on dementia-related fear and maladaptive avoidance, (2) determine the feasibility of the low-cost web-based intervention program, and (3) investigate broad health-related secondary outcomes in older adults.

### Hypotheses

1. We predict greater reductions in dementia-related fear and avoidance (23-item Fear and Avoidance of Memory Loss [FAM-23]) in the REFRAME program group relative to the comparison group.
2. We predict greater improvements in fear of Alzheimer disease, mental health (ie, anxiety), and psychosocial functioning (ie, quality of life, social functioning) in the REFRAME program group relative to comparison.
3. We predict a greater reduction in self-reported subjective memory failures in the REFRAME program relative to comparison.

## Methods

### Inclusion and Exclusion Criteria

Inclusion and exclusion criteria for the study as presented in Textbox 1.

**Summary of inclusion and exclusion criteria.**

**Inclusion criteria**

- Aged 55 or older
- Elevated dementia-related fear as measured by the 23-item Fear and Avoidance of Memory Loss (FAM-23) Scale (score of ≥61).
- Able and willing to provide informed consent.
- Able to read/write in English.
- Willingness to be randomized to the intervention group.
- Willingness to complete 3 weeks of self-guided intervention, questionnaires, and cognitive tests.
- Access to internet for completion of questionnaires and intervention materials.
- Resident in the larger Chicago (Illinois) area.

**Exclusion criteria**

- Diagnosis of mild cognitive impairment, Alzheimer disease, or dementia by a health care provider.
- Impaired cognitive or neurologic function as determined by the Montreal Cognitive Assessment for blind individuals (MoCA BLIND; score of <18 of 22).
- Unstable medical condition (hospitalization in the last 6 weeks or repeated emergency room visits).
- Severe depression (15-item Geriatric Depression Scale [GDS-15] cut-off score of ≥12).
- Undergoing psychotherapy treatment for anxiety or depression.
- Current participation in another psychotherapy.
- Inadequate vision or hearing to interact with study material.
- Current substance use disorder.

### Sample Size

Eighty participants will be recruited (40 in each group). Power was estimated using a mixed ANOVA framework to detect a between-within interaction in G*Power with the following assumptions: medium effect size (*f*)=0.25, Type I error rate=0.05, sample size=80, groups=2, repeated measures=6 (Table 1), correlation among repeated measures=0.5, and nonsphericity correction epsilon=1.0 (ie, no correction) [18]. These assumptions yielded high power (99%). We anticipate losing some participants to follow-up and investigated a nonsphericity epsilon of 0.5; power remained at 92% even with the more conservative epsilon and with n=40. Although these power estimates are high, a goal of this study is to determine the effect sizes (within-subjects and across groups) for future randomized controlled trials.

**Table 1:** Timing of outcome measures and assessments.

Measures	2 weeks before the intervention	Intervention (3 sessions)			1 week after the intervention	Follow-up (4 weeks after the intervention)
		1	2	3		
Demographic (online)	✓^a^					
23-Item Fear and Avoidance of Memory Loss (FAM-23)	✓	✓	✓	✓	✓	✓
Fear of Alzheimer’s Disease Scale (FADS)	✓	✓			✓	
Memory Failure Scale (MFS)		✓			✓	✓
Patient-Reported Outcomes Measurement Information System, 29-item (PROMIS-29)		✓			✓	✓
World Health Organization-Five Well-Being Index (WHO-5)		✓			✓	✓
Montreal Cognitive Assessment for blind individuals (MoCA BLIND)	✓					
15-Item Geriatric Depression Scale (GDS-15)	✓					
Patient Expectation Scale		✓				
1-item Patient Global Impression of Change (PGIC)						✓
Coronavirus Anxiety Scale (CAS)		✓			✓	✓
Qualitative questionnaire						✓

^a^✓ indicates the time points at which measures were taken.

### Recruitment Methods

Participants will be recruited through community-based outreach methods, digital and print advertising (eg, flyers, postcards, email outreach, web postings, advertisements on transit lines, newspaper advertisements, radio advertisements), and official registries (eg, Research Match). One of the study PIs (JG) also has a list of participants from previous research studies that have agreed to be contacted for future projects. If a participant is interested, he/she can complete an online screening survey in REDCap to assess initial eligibility before a longer screening phone call. Potential participants can express their interest to take part by means of direct contact with the researchers via phone or email, after which they would be invited to complete the screening and consent process.

### Consent Process

Because the study will be conducted remotely, we will obtain e-consent from participants via REDCap. Participants will be able to access the consent form as soon as they enter the study and they will only be redirected to the main study if they give their consent. Participants can review the consent form, sign their name, and provide other basic demographic information required if they agree to take part. They will also receive a read-only copy of the consent form, which they can review, download, or print. Participants will be explicitly informed that signing the electronic copy is equivalent to signing a physical document.

### Study Design

The study design is a randomized controlled trial with a between-groups experimental comparison of 2 groups: REFRAME versus psychoeducation plus mindfulness only. The REFRAME program group will participate in psychoeducation, mindfulness, and behavioral activation activities. The comparison group will participate in psychoeducation and mindfulness, but not in behavioral activation activities. Thus, the study will allow us to determine the impact of psychoeducation and mindfulness (within-subjects in the comparison group) on fear reduction, in addition to approach-oriented exercises. The study will consist of 6 time points (2 baselines, 2 midtreatment, after treatment, and follow-up). At each time point, participants will complete outcome questionnaires.

### Measures

Participants will be asked to complete questionnaires 2 weeks before commencing the intervention, at baseline, during the intervention (before Week 2 and Week 3), 1-week after the intervention, and at 4-week follow-up. All assessments will be completed using the REDCap platform with follow-up assessments carried out online via an email link [19]. The primary outcome measure will be the 23-item Fear and Avoidance of Memory Loss (FAM-23) Scale [3], which will assess fear and avoidance of memory loss. This will be administered 2 weeks before commencing the intervention. The Montreal Cognitive Assessment for blind individuals (MoCA BLIND) will also be administered at this time point to screen for cognitive impairment. Secondary outcome measures include the Fear of Alzheimer’s Disease Scale (FADS [20]); World Health Organization-Five Well-Being Index (WHO-5); NIH Patient-Reported Outcomes Measurement Information System, 29-item (PROMIS-29 version 2.0); and Memory Failure Scale (MFS [21]). These assessments will be completed at baseline, after the intervention, and at follow-up. Depression will be measured using the 15-item Geriatric Depression Scale (GDS-15 [22]) 2 weeks after the intervention and at 1-week follow-up. Participants will also complete the 5-item Coronavirus Anxiety Scale (CAS [23]) at baseline, 1-week after the intervention, and at 4-week follow-up. Finally, participants will complete the 1-item Patient Global Impression of Change (PGIC) and brief qualitative interviews at 4-week follow-up. Upon completion of the study, participants will receive a 1-page handout written in lay terms explaining ways to promote brain health, with information for mental health services provided if needed.

### Intervention Overview

The intervention will be delivered over 3 weeks for both groups. The REFRAME group will receive a modular intervention, which is divided into psychoeducation, mindfulness, and behavioral activation. In the first week, participants will learn about concepts such as memory lapses, dementia, fear of memory loss, and causes of fear of memory loss through psychoeducation. Week 1 psychoeducation is divided into 4 modules and participants will be able to complete the modules by listening to the audio clips and completing short online workbook exercises. Week 2 is focused on mindfulness. Participants will learn introductory concepts about mindfulness, meditations (eg, the body scan), noticing thoughts, and grounding through audio and text-based exercises. The final week is focused on delivering behavioral techniques for overcoming avoidance; this involves exercises to increase awareness about avoidance and safety behaviors and identifying ways to challenge them. The comparison group will receive the same intervention during Weeks 1 and 2. For Week 3, they will receive a second week of mindfulness training, which includes additional novel exercises. Audio clips are provided in Multimedia Appendices 1-15.

### Fidelity of the Intervention

Fidelity of the intervention will be checked by reviewing online activities. This will provide a proxy of intervention completion. Finally, postintervention interviews will be used to debrief participants about the intervention.

### Study Team

The intervention was developed by postgraduate students enrolled in an Applied Psychology Master’s Program (PO’L, PP, and JR) under the direct supervision of experienced clinicians (JG, a psychologist, and RS, a geriatrician and behavioral neurologist) and senior researchers in clinical psychology with expertise in mindfulness (MB) and older adult brain health (FF).

## Results

This project received funding from the pilot grant from the Osher Center for Integrative Medicine awarded to FF, JG, and MB in August 2020 and was approved by the Institutional Review Board in Northwestern University Chicago in March 2021. Data collection has commenced as of May 2021 and will continue on a rolling basis until sufficient participants have been recruited.

### Primary Outcomes and Analysis

We will specify a mixed model with a random intercept for each person, as well as fixed effects for treatment (1=REFRAME, 0=otherwise) and 5 dummy codes for the 6 time points. The mixed model will only include treatment–time interactions for the 4 time points after the baseline (for the 2 baselines, treatment should have no effect because the intervention has not yet begun). The primary effects of interest will be the interaction terms for post-treatment and follow-up, tested at α=.05. We will also compare the FAM-23 Scale with baseline using within-subjects *t* tests for all midtreatment, post-treatment, and follow-up time points.

### Secondary Outcomes

The secondary analysis will evaluate if fear of Alzheimer disease, well-being, general anxiety, and memory failures are affected by either intervention. As such, mixed models, similar to above, will be used to analyze scores on the FADS, WHO-5, PROMIS-29, and MFS across time in both groups. In particular, the construct of fear of Alzheimer disease will be examined and compared with the construct of fear of memory loss. The relationship between scores on the FAM-23 Scale and the FADS will be examined through use of a Pearson correlation.

Further information on the data analysis plan is available on the ClinicalTrials registry (Trial Registration Number NCT04821960).

### Participant Debriefing

Upon completion of the study, we will use an open-ended REDCap survey to explore participants’ experiences. We will measure their experience of using the intervention materials, what they found most beneficial, if they plan on continuing to use the skills they have learned, and if they have any recommendations for how we can improve the intervention.

### Missing Data

As per STROBE (Strengthening the Reporting of Observational studies in Epidemiology) guidelines, the total number of participants who take part in each stage of the study will be recorded [24]. Should a participant not take part in a certain wave, reasons will be reported for the absence, if available. Reasons for missing data will be discussed in terms of the intervention group that they were assigned to and the timepoint at which participation ceased. Furthermore, there may be important differences between participants who completed the intervention and those who dropped out. This will be investigated and reported along with the results.

The model does not require complete or imputed data to be estimated. Thus, we anticipate doing an intent-to-treat analysis in which all participants are included in the analysis.

## Discussion

### Limitations

The primary study limitation is the self-guided nature of the intervention. The intervention will be delivered through an online platform comprising weekly audio clips and written passages. This method of delivery could inflate the risk of attrition, as the researchers will not be present while participants engage with the materials. Remote delivery also makes it more difficult to determine if participants comprehended the materials as intended. To mitigate these potential effects, we will measure participants’ engagement with the weekly exercises and questionnaires throughout the study. We will also explicitly ask about accessibility of the materials in the debriefing interviews. A psychologist and geriatrician from the study team will also be available to troubleshoot any issues as they arise. A second limitation is recruitment of the small sample size from a single metropolitan area. Although the sample may limit generalization, the data collected here will help to guide future interventions with larger, more diverse cohorts.

### Strengths and Future Directions

Fear of dementia is associated with a range of negative outcomes including poorer physical health, reduced well-being, and increased perceived memory failures. As such, it is vital that we establish evidence-based interventions to mitigate this fear. Similar to other health anxieties, excessive fear and avoidance of dementia are malleable psychological processes that can be reduced through low-cost interventions. By identifying and effectively treating maladaptive fear early, we may be able to reduce dementia risk, or prevent cases, in later life by fostering healthy lifestyle behaviors (eg, continued cognitive and social engagement). Reducing fear may also encourage people who may be experiencing changes with their memory to seek support from their doctor earlier, which could lead to improved assessment and treatment options.

Future scope of the research will include administering the intervention to a larger, more representative group. In line with the recent push toward early interventions, this research could also be expanded to include midlife (ie, 45+ years). Finally, future work should also aim to identify specific groups who are at a higher risk of experiencing maladaptive fear and avoidance behaviors and design tailored interventions for them; for example, people who are (or have been) a care partner for someone with dementia, or those with a family history of the disease.

## Appendix

Multimedia Appendix 1 Audio file for Week 1 Module 1 of the intervention.

Multimedia Appendix 2 Audio file for Week 1 Module 2.

Multimedia Appendix 3 Audio file for Week 1 Module 3.

Multimedia Appendix 4 Audio file for Week 1 Module 4.

Multimedia Appendix 5 Audio file for Week 2 Module 1.

Multimedia Appendix 6 Audio file for Week 2 Module 2.

Multimedia Appendix 7 Audio file for Week 2 Module 3.

Multimedia Appendix 8 Audio file for Week 3 Module 1 Experimental.

Multimedia Appendix 9 Audio file for Week 3 Module 1 Control.

Multimedia Appendix 10 Audio file for Week 3 Module 2 Experimental.

Multimedia Appendix 11 Audio file for Week 3 Module 2 Control.

Multimedia Appendix 12 Audio file for Week 3 Module 3 Experimental.

Multimedia Appendix 13 Audio file for Week 3 Module 3 Control.

Multimedia Appendix 14 Audio file for Week 3 Module 4 Experimental.

Multimedia Appendix 15 Audio file for Week 3 Module 5 Experimental.

Multimedia Appendix 16 Peer-reviewed report submitted to the Osher Center for Integrative Medicine at Northwestern University for the Osher Pilot Research Awards 2020.

## Abbreviations

CAS: Coronavirus Anxiety Scale
FADS: Fear of Alzheimer’s Disease Scale
FAM-23: 23-item Fear and Avoidance of Memory Loss
GDS-15: 15-item Geriatric Depression Scale
MFS: Memory Failure Scale
MoCA BLIND: Montreal Cognitive Assessment for blind individuals
PGIC: 1-item Patient Global Impression of Change
PROMIS-29: Patient-Reported Outcomes Measurement Information System, 29-item
STROBE: STrengthening the Reporting of OBservational studies in Epidemiology
WHO-5: World Health Organization-Five Well-Being Index
