# Supplementary material for: A Randomized Controlled Trial Investigating the Feasibility of a Low-Intensity Psychological Intervention for Fear of Memory Loss and Quality of Life in Older Adults: Protocol for the Reducing Fear and Avoidance of Memory Loss (REFRAME) Study
Source: JMIR Res Protoc. 2021 Jul 30;10(7):e30514. doi: 10.2196/30514 (PMC8367123; doi:10.2196/30514)
Supplement: Multimedia Appendix 16 [file resprot_v10i7e30514_app17.pdf]

Applicant: James Griffith

Project:	Improving emotional well-being and quality of life in older adults experiencing dementia-related fear
Competition:	Osher Pilot Research Awards 2020
Sponsor:	Osher Center for Integrative Medicine at Northwestern University
Submission deadline:	2020-07-01
Application status:	Complete
Submission created at:	2020-05-19
Submitter:	James Griffith

Application reviews

1. Significance.

Reviewer 1: Strengths • Cognitive decline is a major concern among older adults and the associated fear and anxiety can reduce HRQoL.
Weaknesses • It is not entirely clear that mindfulness vs cognitive-behavior therapy is the best approach to target the fear and avoidance of memory decline.

Reviewer 2: Strengths: - Investigators make a clear justification regarding the negative impact of dementia-related fears on quality of life, cognitive decline, and functional disability in older adults. - Dementia-related fears are understudied in psychological intervention research. There is a need for psychological interventions that specifically target dementia-related fears. - An app-based intervention allows for lower cost, greater accessibility, convenience, and ease of delivery, which allows for a more scalable solution, if demonstrated to be effective. - Propose to develop a tailored mindfulness intervention that specifically targets dementia-related fears.
Weaknesses: - The focus on older adults experiencing sub-clinical levels of dementia-related anxiety is narrow. - The evidence base for conventional meditation, mindfulness, or behavioral-based exercises for fear avoidance, on reducing dementia-related fear in this sample has yet to be established. It may be premature to examine the relative impact of FA-mindfulness training over and above the effects of conventional meditation.

Reviewer 3: Strengths • Quality of life, anxiety, and cognitive functioning are important outcomes for the target population (adults age 55+)
Weaknesses • The evidence regarding the prevalence and impact of dementia-related fear is not well-established

2. Investigator(s).

Reviewer 1: Strengths • The PI has a strong history of grant funding in relevant areas and is well-suited to lead this project. • Co-I Farina brings expertise in aging and dementia • Co-I Bennet brings expertise in mindfulness-based interventions • The team is cross-disciplinary and they have previously worked together.

Weaknesses • None

Reviewer 2: Strengths: - Diversity of expertise is present on this multi-disciplinary team, including expertise in medical social sciences, clinical psychology, neuroscience, and computerized assessments. - Team members are appropriate with strengths in different key areas of the proposed research (Griffith: behavioral interventions, Farina: emotional well-being and quality of life in older adults at-risk of dementia, and Bennet: mindfulness-based training fas a prevention strategy for anxiety and depression). - Team has a history of collaboration and a recent publication on this topic.

Weaknesses: - -No justification for the necessity of 3 MPIs on the proposal. No MPI plan provided outlining the expected roles, organizational structure, and plan for governance for the project. - 2 out of the 3 MPIs are still in training (postdoctoral fellows).

Reviewer 3: Strengths • The team has developed the measure of the primary target of this intervention – fear of cognitive decline • PI is (generally speaking) a well-established and productive researcher • Gerontologist is included as a consultant

Weaknesses • Investigators appear to have much more expertise in measurement science and observational studies than in conducting intervention trials.

3. Innovation.

Reviewer 1: Strengths • The application targets emotional well-being and HRQoL related to dementia-related fears in older adults, which is a relatively unexplored compared to the cognitive domain.

Weaknesses • It is not clear that dementia-related fears are a modifiable risk factor of future consequences.

Reviewer 2: Strengths: - The focus on dementia-related fears is novel - A tailored mindfulness + fear avoidance training program offers a targeted approach for mitigating dementia-related fears and anxiety - Unique focus on the psychological well-being of older adults at risk of dementia, rather than focusing solely on cognitive domains. - App-based delivery of the intervention

Weaknesses: - Given that there hasn't been much research on the efficacy of conventional meditation approaches in this sample, the proposed examination comparing the relative impact of FA-mindfulness training over and above the effects of conventional meditation seems a bit premature.

Reviewer 3: Strengths • Proposal builds on the team's work to develop a new model of fear-avoidance contributing to impaired memory and quality of life in older adults

Weaknesses • None noted

4. Approach.

Reviewer 1: Strengths • The investigators have preliminary data to support the concept that heightened fear and avoidance of memory decline predicts lower HRQoL and increases memory failure. • Use of intent-to-treat analysis and block randomization. • The use of an active meditation only control. • Assessments include both in-person and remote data collection.

Weaknesses • There is very little information about the contents of the intervention or details about the app. • Key details about the inclusion/exclusion criteria are missing. For example, will people with depression and anxiety be included? Psychotropic medications? • Power analysis does not provide justification of the effect size (d=0.4) or consider variability of the outcome measure. • There is no time allocated to develop the app in the study timeline and the budget for developing the app appears to be quite low. • Although an active control group can be a strength, it is not well-justified and unclear if it is matched for attention/contact or what parameters it controls.

Reviewer 2: Strengths: - Overall strategy, methodology and analyses are well-reasoned and appropriate. - Use of an active control group (conventional meditation) in the RCT serves as a strong control condition. - Use of bivariate latent difference modeling to determine whether treatment-related changes in avoidance tendencies facilitate improvements in psychosocial functioning.

Weaknesses: - No justification provided as to whether a 3-week intervention would be sufficient to teach both mindfulness and fear avoidance skills. - Sample size justification assumes a medium effect size of 0.4, which seems to be high given that the comparison group is an active control group (conventional meditation) and the intervention is self-guided, delivered via a mobile app for only 3 weeks. - The primary outcome is measured using a newly developed measure (fear-avoidance of memory decline questionnaire), that only has preliminary support (validation in a sample of N = 67 older adults).

Reviewer 3: Strengths • Randomized controlled pilot trial with an active control group • Objective measurement of intervention adherence (# of audio tracks played and home practices completed are counted within the app) • Semi-structured interview to assess intervention acceptability
Weaknesses • Insufficient detail regarding recruitment; difficult to determine whether enrollment and data collection goals can be achieved within the 1-year project timeline (proposal is to screen N=200 to enroll N=80 for a 3-week intervention, with a 3-month follow-up assessment; inclusion criteria require elevation on fear-avoidance measure developed by the team – has clinical cutoff been established?) • Has the app already been developed? No time is built in for this (nor does it seem possible within 1-year study) • Complications related to international vendor for the intervention app? • A bit more detail regarding the intervention would be helpful – e.g., distinction/similarities between the meditation forms in the control vs. intervention, more detail of the overcoming avoidance exercises, definition of “revision sessions” in week 3 • Including a model (i.e., figure) and consistent, precise use of terms (e.g., rather than broad, overlapping terms such as psychosocial functioning, well-being, quality of life, mental health) would improve clarity of the proposal • is it necessary for participants to complete assessments in-person at pre- and post-intervention? • Exclusion of adults with MCI (via MoCA cutoff 26 of 30) leaves little room for improvement on this measure as a secondary outcome

5. Environment.

Reviewer 1: Strengths • The environment is suitable to carry out this project, most of which will be conducted on-line.
Weaknesses • No major weaknesses.

Reviewer 2: Strengths: - The environment and available resources to support the project are strong. - Consultation with Dr. Shapiro (UIC Gerontologist) and the Alzheimer’s Association of America should support successful recruitment for this project.
Weaknesses: - None.

Reviewer 3: Strengths = robust research department at NU (PI's primary appointment in MSS)
Weakness = limited info regarding co-investigators' institutional environment

Overall review comments

Reviewer 1: In this proposal, the investigators propose to conduct an RCT to test FA-mindfulness training vs meditation in 80 older adults. In general, this application is responsive to the RFA and there is innovation in addressing a research gap in the emotional domain (i.e., fear, avoidance) associated with memory decline. The investigators are excellent and strengths with the approach include promising preliminary data and the use of an active control condition. Weaknesses include some key missing details about the contents of the app-based intervention and control, unclear inclusion/exclusion criteria, and concerns that the study will be under-powered to detect differences between the two arms. In addition, there are concerns about the feasibility of developing and testing the app program within the budget and timeline. The overall impact of the project is moderate.

Reviewer 2: Dementia-related fears are common among older adults and can lead to vicious cognitive-behavioral cycles that have negative consequences for their health and well-being. The proposed project aims to develop an a tailored, app-based, mindfulness + fear avoidance training program (FA-mindfulness) for mitigating dementia-related fears and improving well-being in a sample of older adults experiencing heightened

dementia-related fear. The investigators propose to conduct a randomized controlled study comparing the tailored FA-mindfulness program with an active control (conventional meditation program). They will examine adherence, dementia-related fear and avoidance, mental health, and psycho-social functioning. Strengths of the application include a novel focus on dementia-related fears (an understudied area in psychological intervention research), the use of an active control group (conventional meditation), and app-based delivery of the program. Weaknesses include the lack of justification for whether a 3-week self-guided intervention would be sufficient to teach mindfulness and fear avoidance skills to older adults experiencing heightened dementia-related fear. Moreover, given this topic and this sample tend to be understudied, there has been minimal research supporting the use of conventional meditation, mindfulness training, or behavioral-based exercise for fear avoidance for reducing dementia-related fear in this sample or the use of the fear-avoidance of memory decline questionnaire. Overall, I am enthusiastic about this proposal and I believe this program of research has the potential to have a positive impact on the research community and older adults experiencing heightened fear of dementia. This research can provide the preliminary data to support a larger grant submission to the National Institute of Aging.

Reviewer 3: This proposal seeks to conduct a pilot trial of an app-based mindfulness + overcoming avoidance intervention vs. conventional mindfulness meditation for adults age 55+. Strengths include: novel theoretical basis, active control group in a randomized trial, some elements of the measurement approach, sophisticated modeling/analytic techniques, and a relatively strong investigative team with established collaboration. Enthusiasm for the proposal is tempered primarily by questions about the feasibility of the approach within the 1-year project timeline and lack of clear detail regarding the intervention components.

Application scores

- Overall score: 3.33
  (4,2,4)
